# Expression of HCA2 Receptors in Femoral Epiphysis and Metaphysis of Rats with Dexamethasone-Induced Osteoporosis

**Published:** 2016-06-19

**Authors:** Tahoora Shomali, Mohammad Kamalpour, Mehdi Fazeli, Alireza Rafati

**Affiliations:** *Division of Pharmacology and Toxicology, Department of Basic Sciences, School of Veterinary Medicine, Shiraz University, Shiraz, Iran.*

**Keywords:** Glucocorticoid-induced osteoporosis, hydroxyl-carboxylic acid receptor 2, RT-qPCR, immunohistochemistry, rat

## Abstract

The present study describes the changes in expression of hydroxy- carboxylic acid receptor 2 (HCA2 receptor) in femoral epiphysis and metaphysis of rats with glucocorticoid-induced osteoporosis (GIO). 16 growing male Sprauge dawley rats were randomly divided into two equal groups consisting of normal control and rats that were rendered osteoporotic by receiving 0.1 mg/kg/day dexamethasone subcutaneously. After 4 weeks, all rats were sacrificed and immediately right and left femoral bones were removed for RT-qPCR and histological examination, respectively. Immunohistochemical parameters using a primary rabbit polyclonal GPR109A antibody in hematoxylin and eosin- counter stained slides were determined. HCA2 receptor expression was evaluated using RT- qPCR. Qualitative and histomorphometric evaluation of slides revealed the establishment of glucocorticoid- induced osteoporosis (GIO) in rats treated with dexamethasone. In immunohistochemical study, dexamethasone administration appreciably reduced receptor density in all evaluated cell types and in total slides as compared to control. mRNA level of HCA2 receptor gene was reduced in dexamethasone- treated group. GIO may be associated with down regulation of HCA2 receptors in proximal femoral bone of rats at mRNA as well as protein level in no- cell type-specific manner, although reduction in protein expression needs to be further confirmed by western blotting.

Glucocorticoids (GCs) as potent anti-inflammatory and immunosuppressive agents are used in myriad conditions for more than half century. Unfortunately, this class of drugs is notorious for a diverse array of side effects including glucocorticoid- induced osteoporosis (GIO) which is the most prevalent form of secondary osteoporosis (1). Osteoporotic fractures are one of the most devastating and money-consuming consequences of GC therapy especially in long term and/or high dose regimens, and affect 30-50% of patients (2-4).

Different mechanisms are proposed for GIO including decreased bone remodeling and a state of low bone turnover with trabecular bone loss and damaged bone microarchitecture, as the most significant features of affected bones (5).

The primary action of GCs is on osteoblasts, where by decreasing replication and impairing differentiation and maturation, may lead to decreased bone formation (6, 7). Osteocytes are also affected (8) and increased bone resorption is observed in the early phase of GC treatment (9). It is believed that disruption of bone marrow microcirculation and increased adipogenesis contribute to GIO development, although currently no available anti-osteoporosis agent specifically targets microcirculation and bone marrow adipogenesis (10).

In a microarray analysis study performed by Yao et al., 2008, GC excess in mice was associated with early activation of genes associated with osteoclastogenesis and adipogenesis and a later suppression of genes associated with osteogenesis and mineralization (11).

The hydroxy-carboxylic acid receptor 2 (HCA2 receptor) is a G-protein coupled receptor (GPCR) and serves as the target of antidyslipidemic drug nicotinic acid (or niacin) (12-14) a drug which has been in use since the 1950’s (15) and is still the most efficacious drug approved to raise HDL cholesterol plasma levels (16). These receptors are predominantly expressed on adipocytes and mediate the inhibition of lipolysis by coupling to G_i_–type proteins (17). Adipocytes can "sense" metabolic changes in the environment by these cell surface receptors and respond through lipolytic regulation (18).

To better elucidate the possible relationship between lipid homeostasis and GIO, the present study describes the changes in expression of HCA2 receptors in femoral epiphysis and metaphysis of rats with GIO at both mRNA and protein levels by using quantitative real-time polymerase chain reaction (RT- qPCR) and immunohistochemical methods.

## Materials and methods

**Study design**

Sixteen growing male Sprauge dawley rats with a mean body weight of 150 g were randomly divided into two equal groups consisting of normal control group (CG) that was left without drug treatment, and osteoporotic group that received 0.1 mg/kg/day dexamethasone sodium phosphate (Aburaihan Co, Iran) injected subcutaneously as described by Ferretti et al. (19). After 4 weeks, all rats were sacrificed under deep ether anesthesia and immediately right and left femoral bones were removed for RT-qPCR and histological examination, respectively. RT-qPCR samples were kept in -70ºC until use. Rats had free access to commercial chew pellets and tap water. The ambient conditions were temperature about 23° C and a 12h/12h, light/dark cycle.

All procedures used in the present study are in accordance with Shiraz University ethical guidelines for care and use of animals in experiments which are compatible with European convention for the protection of vertebrate animals used for experimental and other scientific purposes.

**Histomorphometric study**

Hematoxylin and eosin (H&E)-stained slides from proximal epiphysis and metaphysis of left femoral bones were blindly evaluated qualitatively and quantitatively for histomorphometric param-eters including epiphyseal trabecular width (epi. Tb.Wi), metaphyseal trabecular width (Mt. Tb.Wi), epiphyseal bone area/ tissue area (epi. B.Ar/T.Ar) and metaphyseal bone area/ tissue area (Mt. B.Ar/T.Ar) by using Ziess Axiovision LE 4.8.2 software. All parameters were measured in a central zone of epiphysis or secondary spongiosa in metaphysis. The mean value of ten measurements of each parameter in each sample was calculated. The nomenclature of parameters is in compliance with American society of bone and mineral research (ASBMR) histomorphometry nomen-clature committee (20).

**Immunohistochemical evaluation**

Four μm-paraffin sections from proximal epiphysis and metaphysis of left femoral bones were obtained in median plate after formic acid-sodium citrate decalcification. Endogenous peroxidases were inactivated by 3 minute incubation in 3% hydrogen peroxide in methanol. The primary antibody for immunohistochemical staining was rabbit polyclonal GPR109A antibody (1:300, incubation for 60 min at room temperature) which reacts with rat HCA_2_ receptor, prepared by **Biorbyt Ltd.**, United Kingdom. Visualization was made by EnVision™ detection systems peroxidase/ diaminobenzidine (DAB) for rabbit/mouse antibodies (1:2, 30 and 5 min incubation at room temperature for secondary antibody and DAB chromogen, respectively) (Dako, Denmark). Then, slides were counter stained with hematoxylin. Negative controls were treated with secondary antibody only. Fat tissue was used as a positive control for presence of the receptor.

Color images of the slides under light microscope were prepared and blindly evaluated by Softonic^®^ image analyzer software with regard to hue, saturation and brightness (HSB), which is inversely correlated to receptor density (21). For quantitative determination of cellular density of the receptor, at least 5 cells of each type in each slide were randomly assayed and the mean HSB value was calculated. HSB value of the total slide (including bone marrow and trabeculae) was also determined.

**RNA isolation and RT-qPCR**

Samples weighing 50-100 mg, from proximal epiphysis and metaphysis of right femoral bones (including bone marrow and trabeculae) were powdered manually in liquid nitrogen and total RNA was extracted using *AccuZol*™ total RNA extraction solution (Bioneer, Korea), as described by manufacturer and treated with DNase I (Fermentas Inc., United States). First strand cDNA was synthesized from 8 μl of total RNA by using *RocketScript*^TM^ cycle RT premix (Bioneer, Korea). A reverse-transcriptase PCR was performed before the RT-qPCR for confirmation of the presence of HCA2 receptor gene in samples. The RT-qPCR contained 3 μl of cDNA, 0.5 μl of 250 nM forward and reverse primers, 6.25 μl of AccuPower*® *GreenStar^TM^ *qPCR premix* (Bioneer, Korea) that included SYBR Green I dye and 2.25 μl distilled water using MJ Mini™Thermal cycler and MiniOpticon™ RT-qPCR system (Bio-Rad, United States). The following primers were used: GPR109A/HCA2, forward: 5´-CGGTGGTCTACT-ATTTCTCC-3´, reverse: 5´-CCCCTGGAATA-CTTCTGATT-3´; Glyceraldehyde 3-phosphate dehydrogenase (GAPDH), forward: 5´-AAGGA-TACTGAGAGCAAGAG-3´; GAPDH, reverse: 5´-TGATGGTATTCGAGAGAAGG-3´, which yiel-ded 159 and 154 bp products, respectively. . The RT-qPCR thermocycling conditions were as follows: 94ºC for 30 seconds (initial denaturation), 94°C for 5 seconds (denaturation) and 55°C for 30 seconds (annealing), plate read in 40 cycles. Melting curves were produced for HCA2 receptor and GAPDH PCR products. Quantification was performed by the comparative Ct method with GAPDH used as internal control. Moreover, a PCR reaction on extracted RNA with HCA2 receptor primers was performed to rule out genomic DNA contamination.

**Statistical analyzes**

Data were presented as mean±SD and analyzed by two independent samples Mann-Whitney U test except for the result of RT-qPCR that was analyzed by independent samples t-test. A significance level of p<0.05 was considered for all comparisons.

## Results

**Confirmation of GIO by histomorphometric**

**evaluation**

**Fig 1:**
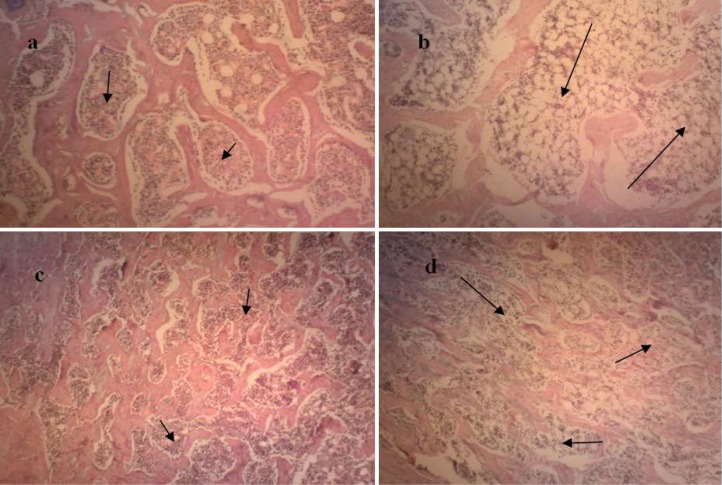
Representative photomicrographs of epiphysis (a, b) and metaphysis (b, c) of proximal femur in control (a, c) and dexamethasone-treated rats (b, d) (H&E staining, mag. 64). Trabeculae occupy less portion of the tissue in dexamethasone-treated rat as compared to control without a change in connectivity which demonstrates osteoporosis. Arrows show the spaces between trabeculae in different groups.

Qualitative evaluation of slides revealed the presence of GIO in rats treated with dexamethasone with more marrow spaces between thinned trabeculae without a change in their connectivity (Fig. 1). In quantitative evaluation, all measured histomorphometric parameters were significantly lower in dexamethasone-treated group as compared to control (p<0.05). Data are presented in Table 1.

**Immunohistochemical evaluation of receptor protein expression**

**Fig 2:**
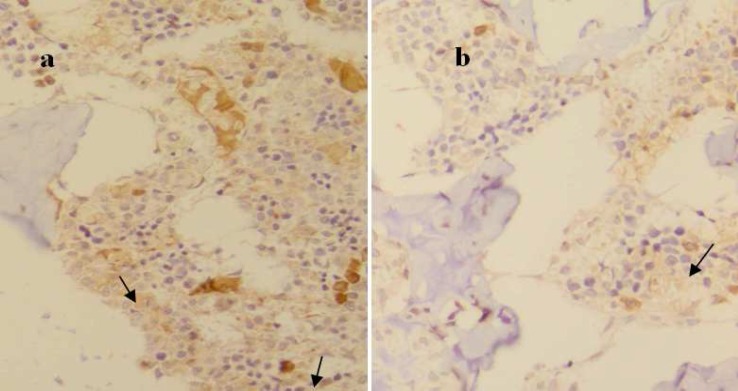
Representative photomicrographs of epiphyseal marrow of proximal femur in control (a) and dexamethasone-treated rats (b) (immunohistochemical staining for HCA2 receptor, mag. 64). Arrows show different color intensity of some marrow cells.

Table 2 shows HSB values of different cells in control and dexamethasone-treated rats. Dexa-methasone administration appreciably reduced receptor density in all cell types and in total slide compared to control which was demonstrated by higher HSB values in rats with GIO irrespective to cell type (p<0.05) (Fig. 2). Due to scarcity of osteoclasts in the histological slides, we were not able to quantify receptor density in this type of cell, although they were positive for receptor expression.

**Evaluation of HCA2 receptor mRNA expression by RT-qPCR**

RNAs isolated from femoral samples were used for RT-qPCR to determine whether mRNA transcripts encoding HCA2 receptors were present in analyzed samples.

**Table 1:** Trabecular bone histomorphometric parameters

groups	Control group	Dexamethasone-treated group	P-value
parameters			
epi. Tb.Wi (µm)	86.2± 14.9	60.3± 12.2	0.002
Mt. Tb.Wi (µm)	40.8± 6.2	28.9± 2.81	≤0.001
epi. B.Ar/T.Ar (%)	32± 6.0	21.4± 4.05	0.002
Mt. B.Ar/T.Ar (%)	35.6± 5.7	20.9± 4.21	≤0.001

Mean± SD are presented for control and Dexamethasone-treated rats (n= 8 each). All parameters were significantly lower in dexamethasone-treated group as compared to control (P<0.05). epi. Tb.Wi: epiphyseal trabecular width; Mt. Tb.Wi: metaphyseal trabecular width; epi. B.Ar/T.Ar: epiphyseal bone area/tissue area and Mt. B.Ar/T.Ar: metaphyseal bone area/tissue area.

**Table 2:** Cellular density of HCA2 receptor in different cells based on hue, saturation, brightness (HSB) value.

**Groups**	**Control group**	**Dexamethasone-** **treated group**	**P-value**
**Cell type**			
Marrow cells	83.6± 11.5	125± 24.8	0.008
Chondroblasts	149± 13.1	187± 2.80	0.008
Chondrocytes	166± 5.34	185± 1.66	0.008
Osteoblasts	141± 1.29	187± 2.81	0.008
Osteocytes	143± 1.29	189± 1.39	0.008
Fibroblasts	136± 10.8	193± 3.46	0.008
Adipocytes	130± 13.0	180± 22.5	0.016
Vascular endothelial cells	128± 5.65	166± 4.65	0.008
Striated muscle cells	139± 2.14	194± 1.03	0.008
Total slide	147± 9.02	193± 9.54	0.001

Mean± SD are presented for control and Dexamethasone-treated rats. HSB value is inversely correlated with receptor density.The groups were significantly different in all parameters (P< 0.05).

Relative mRNA level of HCA2 receptor gene as determined by 2^-∆∆Ct^ method was 2.46±0.311 in control group and 0.579±0.115 in dexamethasone-treated group which were significantly different with P= 0.001. Single product-specific melting temperatures were 85.2 ºC and 85.6 ºC for HCA2 receptor and GAPDH, respectively.

## Discussion

GCs show a fascinating and versatile array of complex physiological and pharmacological effects which are at least in part mediated through transcriptional regulation by the glucocorticoid receptor (GR) in a way that activation of GR results in tissue-specific changes at gene expression level with some genes being activated whereas others are repressed (22). In the present study, we observed that GIO can affect HCA2 receptor expression in different cells of epiphysis and metaphysis of rat femoral bone in a way that GC administration resulted in a decrease in receptor expression at both mRNA and protein levels. The decrease in receptor density due to GC administration was not cell type-specific and encompassed all evaluated cell types including bone cells (osteoblasts and osteocytes) and cartilage cells as well as adipocytes, fibroblasts, vascular endothelial cells and striated muscle cells. It should be mentioned that due to low number of osteoclasts in histological sections we were not able to quantify receptor density in this cell type.

Factors that regulate the expression of HCA2 receptors are not fully elucidated, although some of them were described in previous studies. For example, interferon gamma was shown to enhance HCA2 receptor expression in macrophages (23) and mRNA expression of this receptor has been increased in mature human and murine adipocytes as well as *in vivo* in epididymal fat pads of mice by using rosiglitazone (a peroxisome proliferator-activated receptor gamma (PPAR-γ) agonist) (24). More recently, it has been shown that lipopolysaccharide (LPS) increases HCA2 receptor expression in RAW 264.7 macrophages (25). Moreover, both LPS and TNF alpha up regulate HCA2 receptors in adipocytes (18, 26). In a study by Feingold et al., LPS, TNF alpha, and interleukin (IL) 1 increased HCA2 receptor expression in adipose tissue; the authors concluded that inflammation is an enhancing factor for HCA2 receptor expression (27). Expression levels of inflammatory genes such as IL-6 and TNF alpha is high in bone marrow adipocytes (28). Since GCs suppress production of inflammatory mediators like TNF alpha (29), reduced expression of HCA2 receptors as observed in our study seems quite plausible.

In GIO, the most dramatic adverse effect of GC excess is directly on the bone cells, especially osteoblasts (30), although the increased adiposity of bone marrow is also important

G Cs reduce the production of new osteoblast precursors and cause early apoptosis of the mature, matrix-secreting osteoblasts (31). Inadequate numbers of osteoblasts and incomplete erosion cavity repair during bone remodeling are the main causes of the reduction in cancellous bone area, wall width, trabecular width and bone formation rate typically found in GIO (31).

GCs reduce osteoblast differentiation by attenuating Akt (protein kinase B) phosphorylation (32). On the other hand, it has been clearly demonstrated that niacin activates the PI3K/Akt cascade by acting on HCA2 receptors (33). In the present study, GCs administration reduced HCA2 receptor expression in osteoblasts. This reduction in receptor density and its consequent reducing effect on PI3K/Akt cascade activation may contribute to the attenuating effect of GCs on Akt activation and reduced osteoblast differentiation which is frequently observed in GIO, although this needs to be examined properly

On the other hand, bone marrow adiposity increases in age-related osteoporosis and there is an inverse relationship between bone mass and bone marrow adiposity (34-36). This feature of age-related osteoporosis is very similar to that of GIO where increased adipogenesis potentiates GIO development. Consistent with our findings, in a study performed by Liu et al., expression of HCA2 receptors in bone marrow adipocytes of mice showed a significant decrease with age (28). These findings may show a role for HCA2 receptors in accelerated adipogenesis observed in both GIO and age-related osteoporosis.

In conclusion, GIO may be associated with down regulation of HCA2 receptors in proximal femoral bone of rats at mRNA level as well as protein expression in no cell type-specific manner, although reduction in protein expression needs to be further confirmed by western blotting.
